# Autoimmune Hepatitis with Acute Presentation: Clinical, Biochemical, and Histological Features of 126 Patients

**DOI:** 10.1155/2022/6470847

**Published:** 2022-09-26

**Authors:** Álvaro Urzúa, Carolina Pizarro, Abraham Gajardo, Rafael Poniachik, Claudia Pavez, Máximo Cattaneo, Javier Brahm, Laura Carreño, Jaime Poniachik

**Affiliations:** ^1^Hospital Clínico Universidad de Chile, Department of Internal Medicine, Section of Gastroenterology, Santiago, Chile; ^2^Gastroenterology Section, Clínica Santa María, Santiago, Chile; ^3^Hospital Clínico Universidad de Chile, Intensive Care Unit, Santiago, Chile; ^4^Hospital Clínico Universidad de Chile, School of Medicine, Santiago, Chile; ^5^Clínica Las Condes, Department of Gastroenterology, Santiago, Chile; ^6^Hospital Clínico Universidad de Chile, Department of Pathology, Santiago, Chile

## Abstract

**Introduction:**

Autoimmune hepatitis (AIH) is a chronic liver disease with a relevant inflammatory component and an unknown etiology. Evidence for clinical characteristics and risk factors in large cohorts of patients with acute AIH (AAIH) is lacking. We clinically characterized patients with AAIH, the prevalence of a combined adverse outcome (death or liver transplantation (LT)), and its risk factors.

**Methods:**

A retrospective study of adult patients diagnosed with AAIH at three centers (Santiago, Chile; 2000–2018) was conducted. Clinical and laboratory characteristics were obtained. A liver biopsy was performed for all patients. Descriptive statistics and logistic regression models were used.

**Results:**

A total of 126 patients were admitted; 77% were female, 33 (26.2%) had a severe presentation, and 14 (11.1%) had a fulminant presentation. Overall, 24 patients (19.0%) lacked typical autoantibodies, and 26.2% had immunoglobulin G levels in the normal range. The most frequent histological findings were plasma cells (86.5%), interface hepatitis (81.7%), and chronic hepatitis (81.0%). Rosettes were uncommon (35.6%). Advanced fibrosis was present in 27% of patients. Combined adverse outcomes occurred in 7.9% of cases, all fulminant with histological cholestasis. Alkaline phosphatase, bilirubin, and prothrombin less than 50% were independent risk factors for in-hospital death or LT (*p* value <0.05). Although corticosteroid treatment was associated with better outcomes (OR 0.095, *p* value = 0.013), more severe patients were less likely to receive this therapy. *Discussion*. In this large cohort of patients with AAIH, clinical characteristics differ from those reported in patients with chronic AIH. Fulminant hepatitis, histological cholestasis, alkaline phosphatase, bilirubin, and prothrombin were associated with death/LT.

## 1. Introduction

Autoimmune hepatitis (AIH) is a chronic inflammatory liver disease of unknown etiology characterized by the presence of autoantibodies, hypergammaglobulinemia, and interface hepatitis [1]. It has been classically described in Caucasian and young women; however, it has currently been shown to affect individuals of both sexes and of all ages [2, 3].

The clinical presentation of AIH is heterogeneous, ranging from asymptomatic to acute fulminant hepatitis. It is estimated that between 25 and 75% of patients with AIH will present with the acute form [4], a group that includes patients with *true* acute AIH (AAIH) and those with exacerbations of an undiagnosed preexisting AIH. In the American and European series, 3 to 6% of patients with AAIH presented a fulminant form [4–6], defined by the presence of an international normalized ratio (INR) > 1.5–2 and encephalopathy [7], with a low transplant-free survival rate.

On the other hand, previous evidence suggests that in AAIH, clinical and histological findings typically described in chronic AIH could be less frequent. Patients frequently have undetectable autoantibodies at the time of the AIH diagnosis, which may occur in up to 39% of the cases, and normal IgG levels have been reported to occur in 25 to 39% of individuals [8, 9]. The most common findings reported in patients with AAIH are centrilobular necrosis in 80 to 90% and lymphoplasmacytic infiltrate in 50 to 90% of patients, while interface hepatitis is evident in only 50 to 70% of patients [10, 11]. Consequently, evidence for traditional diagnostic criteria proposed by the international AIH group that had been validated, including both the original and the simplified score in clinical practice for chronic AIH cohorts [12], are lacking in the acute setting, and they have not been widely validated in these patients [13–15]. Similarly, risk factors for adverse outcomes in patients with AIH have been scarcely reported in large patient cohorts.

Considering the particular serological and histological features of AAIH, its early diagnosis continues to be challenging. The objective of this study is to describe the clinical, laboratory, and histological features of AAIH and to report the mortality and need for liver transplant (LT), together with their risk factors in these patients.

## 2. Patients and Methods

### 2.1. Study Population

This retrospective study included all patients older than 15 years old discharged between 2000 and 2018 with a diagnosis of AAIH from one of three tertiary hospitals located in Santiago, Chile: Hospital Clínico Universidad de Chile, Clínica Santa María, and Clínica Las Condes. AAIH was suspected in patients with clinical features of acute hepatitis, with no evidence of pre-existing liver disease and symptom onset < 26 weeks. In addition, transaminases were greater than 10 times the upper limit and/or total bilirubin greater than 10 mg/dL. Liver biopsy was performed in all patients; other causes of acute hepatitis, such as viral, drug-induced, metabolic, and ischemic, were ruled out by clinical features and history and serology, molecular biology, and imaging testing.

Three trained physicians reviewed clinical records to obtain clinical, laboratory, and histological data, as well as the presence of in-hospital outcomes (death or LT).

### 2.2. Clinical and Laboratory Evaluation

Laboratory tests were performed at hospital admission to determine liver enzymes, including alanine aminotransferase (ALT), aspartate aminotransferase (AST), gamma-glutamyl transferase (GGT), alkaline phosphatase (AP), total bilirubin and prothrombin time (PT), with the International Normalized Ratio (INR). The presence of antinuclear antibodies (ANAs), anti-smooth muscle antibodies (SMAs), anti-liver-kidney microsomal type 1 antibodies, and antimitochondrial antibodies (AMA) were assessed in plasma samples from all the included patients and analyzed using indirect immunofluorescence, with titers greater than 1 : 40 considered positive. During the study period, immunoblotting for testing antibodies (liver panel) was not available in our country; and thus, antisoluble liver antigen/liver-pancreas (SLA/LP) antibodies and liver cytosol-specific antibody type 1 (anti-LC1) were not evaluated. IgG was assessed in all but 4 patients. Other potential markers of AIH (anti-LKM-1 antibody, antimitochondrial antibodies) were assessed in a subgroup of patients according to the treating physician's criteria. Sex, age at diagnosis, and the coexistence of autoimmune diseases were also obtained.

### 2.3. Histological Evaluation

Liver biopsy was performed in all patients (123 were performed via a percutaneous approach, two by the transjugular route, and one in the explanted liver). The histological samples were examined by expert pathologists to evaluate liver necrosis and its degree, interface hepatitis, fibrosis stage, and inflammatory infiltrate characteristics. For fibrosis staging, the METAVIR scale was used, since it is better known and understood by clinicians than other scoring systems. The presence of rosettes and cholestasis was also documented.

### 2.4. Scores for AIH

The original score for AIH [16] and the simplified score [17] were assessed in patients with information available to calculate both scores.

### 2.5. Ethical Statement

The study was approved by the research ethics committee of each participating center and followed the tenets outlined in the Declaration of Helsinki and local regulations regarding research in humans.

### 2.6. Disease Severity, Corticosteroid Use, and Outcomes

According to the clinical presentation at hospital admission and based on previously published definitions [15], patients were classified into three groups according to the INR and presence of encephalopathy: acute nonsevere (INR < 1.5), acute severe (INR > 1.5 without encephalopathy) and fulminant (acute severe with hepatic encephalopathy occurring within less than 26 weeks from the onset of jaundice).

The corticosteroid dosing and route of administration were determined based on local guidelines; oral prednisone or intravenous hydrocortisone were the most commonly used. Liver transplant decisions were made by a multidisciplinary team in patients with fulminant AIH or severe AIH who did not respond to steroids. In-hospital death was obtained from discharge records. Liver transplantation or death was considered the primary outcome.

### 2.7. Statistical Analysis

The data were expressed as absolute values and percentages for categorical variables, while quantitative variables were expressed as the mean ± standard deviation or median (p25–p75), as appropriate. Normal data distribution was evaluated using the Shapiro–Wilk normality test. Descriptive statistics were used to report the clinical, laboratory, and histological characteristics of the patients. Differences between patients with and without the primary outcome were determined by Student's *t*-test, the Mann–Whitney test, or Fisher's exact test. Logistic regression was performed to determine unadjusted risk factors for in-hospital mortality/LT. Variables with strong statistical evidence as risk factors (*p* value < 0.05) were included in multivariate models to adjust for prothrombin (less than 50%), except for the latter, which was adjusted for bilirubin. The a priori sample size (*N* = 114) was estimated for a primary outcome prevalence of 8 ± 5%, with a 95% confidence interval. Thus, considering the number of events (primary outcome) in the sample, only two independent variables were included in multivariate models to achieve 5–10 events for each variable [18]. All analyses were performed with Stata version 12.0 (TX: StataCorp LP).

## 3. Results

### 3.1. Clinical Characteristics

A total of 126 patients were admitted by AAIH during the study period. The mean age was 49 ± 15 (range 15 to 85) years, and 77% were women (female: male ratio = 2.3 : 1) (Table 1). A total of 23% had a history of at least one previously diagnosed autoimmune disease, with hypothyroidism being the most prevalent (15%). Seventy-nine patients (63%) presented with nonsevere acute AIH, 33 (26%) with severe, and 14 (11%) with fulminant AIH (Table 1).

The baseline characteristics of patients with and without the primary outcome (in-hospital death or LT) are shown in Table 2. Whereas age, sex, and autoimmune comorbidities were similar between both groups, all patients who died or required LT had a more severe presentation (*p* value <0.001), which was fulminant in all deceased/LT patients.

### 3.2. Laboratory Features

Most patients with AAIH presented with elevated levels of bilirubin and abnormal liver enzymes (Table 1). Bilirubin levels were approximately twice as high in patients who died or received LT (*p* value < 0.0001), and AP was also significantly increased in this group (*p* value = 0.026). The prothrombin percentage was significantly lower (*p* < 0.001). No differences were seen in transaminase (ALT and AST) or GGT levels.

ANA was present in 74% and SMA in 48% of the patients, and both antibodies were negative in 26 patients (21%). AntiLKM-1/AMA was positive in less than 5% of the patients. Immunoglobulin G was above the normal range in 70% of patients. None of these immunological markers differed statistically between patients with or without death/LT (Table 2) or between nonsevere, severe, or fulminant presentations (Table 1).

### 3.3. Histological Findings

The most frequent findings in liver biopsy analysis were plasma cell infiltration (87%), interface hepatitis (82%), and chronic hepatitis (81%). In fact, 89 patients (71%) had the three previous findings together. In contrast, rosettes—a typical histological finding of AIH—were present only in 44 (36%) patients. All the aforementioned were not different between groups (Table 3).

Centrilobular necrosis was moderate or severe in 60% of patients, and 27% had advanced fibrosis (*F*3-4). None of these histological findings were different between those who died or received LT and those who did not (Table 2). However, severe centrilobular necrosis was significantly higher in severe and fulminant presentation (*p*=0.0002) (Table 1). On the other hand, cholestasis was present in 52 (41%) patients in the overall cohort and in all patients who died or underwent transplantation (Table 3).

### 3.4. Diagnostic Score Applicability

**Figure 1:**
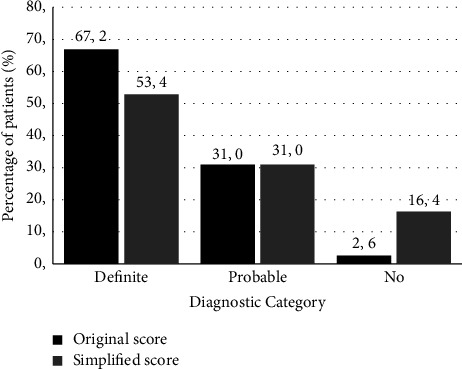
Performance of the original and simplified scores in identifying 117 patients in the cohort.

The original score and simplified score for AIH were calculated in 117 patients in which all the required information was available. Whereas the original score indicated definite AIH in 78 (67.2%) of the patients, the simplified score was definite in only 62 patients (53.4%). On the other hand, 2.6% and 16.4% of patients scored as having neither definite nor probable hepatitis in both scoring systems, respectively (Figure 1).

### 3.5. Liver Transplantation, In-Hospital Mortality, and Their Risk Factors

In-hospital death or LT (primary outcome) occurred in ten patients (7.9%): nine underwent transplantation (3 died), and 1 died without LT. Thus, in-hospital mortality was 3.2% in the overall cohort and 33% in transplanted patients. The characteristics of the deceased patients are shown in Table 4.

All 10 patients who died or required LT had fulminant hepatitis. Of the 14 patients from the fulminant group, 64% required liver transplantation, and 29% died; 71% met the primary outcome. The absence of fulminant hepatitis perfectly predicted the primary outcome, and therefore, disease severity could not be included in regression analyses. Similarly, the prevalence of histological cholestasis was 100% in patients with the outcome, so it was also not included in regression analyses. However, both disease severity and histological cholestasis were associated with the outcome (Tables 2 and 3, respectively).

Table 5 shows the factors associated with death/LT in the regression analysis. Alkaline phosphatase, total bilirubin, and prothrombin time (%) increased the odds of death or LT. Conversely, corticosteroid treatment was statistically associated with a lower odds of death/LT. Nevertheless, patients with severe or fulminant hepatitis were less likely to receive corticosteroid treatment (odds ratio (OR) = 0.088, *p* value = 0.026). All these factors and other patient characteristics are summarized according to disease severity in Tables 1 and 6.

## 4. Discussion

In this retrospective study, we describe the clinical, laboratory, and histological characteristics of AAIH and identify disease severity, histological cholestasis, alkaline phosphatase, bilirubin, and prothrombin as risk factors for in-hospital death/LT. Previous findings show that approximately 40% of patients will present with AAIH, and 3 to 6% will present with fulminant AIH, a subgroup with high morbidity and mortality rates [19]. As AAIH has a different clinical presentation than chronic hepatitis [9], prompt diagnosis and the identification of risk factors for adverse outcomes are challenging for early therapeutic decision-making.

The clinical and demographic characteristics of our cohort were similar to previously published studies on patients with AAIH [10, 11], and to the best of our knowledge, the present cohort represents one of the largest published series to date. We confirmed that the female sex is the most frequent in this type of presentation, the same as chronic forms of presentation. Regarding the age of presentation, most patients were diagnosed between their thirties and fifties, but with a wide range of age at onset (15 to 85 years). Patients over 65 years old represent 13.5% of our cohort; in older patients with icteric hepatitis, autoimmune hepatitis should be ruled out. This is somehow different from previous reports, in which autoimmune hepatitis in the elderly is frequently asymptomatic [20]. The concurrent autoimmune disease was present in 23% of patients, with hypothyroidism (15%) the most frequent disease, consistent with chronic forms of presentation of the disease [7].

Laboratory findings in AAIH are similar to those in other acute forms of hepatitis but different from those in chronic AIH. Consequently, in our cohort, most of the patients had a picture of acute hepatitis with elevated transaminases and bilirubin with or without prothrombin time alteration. Unlike AIH with a nonacute presentation, transaminase levels tend to increase more significantly if the presentation is acute [21]. This was also seen in our cohort for both ALT and AST. In chronic AIH, the presence of autoantibodies (ANA and AML) and elevated plasma levels of IgG are typically described. In fact, these findings are considered in both scores proposed for diagnosis [16, 17]. However, as previously reported [6, 10, 11], these findings may be absent in patients with AAIH, as observed in our cohort, which may affect the accuracy of the diagnosis. Although ANA was the most frequent autoantibody, it was present in only 75% of the patients. Moreover, 21% of them did not have any classical AIH antibodies. Furthermore, IgG levels were elevated in only 2/3 of the patients, similar to previous reports [10]. Other second-level serological tests for autoantibodies detection were not done, since they were not available in our country at the time of the study period inclusion. This is an important limitation, since some second-level autoantibodies that can be very helpful for diagnosis, such as anti-LKM1, anti-SLA, and anti-LC1, could be detected only thanks to high-sensitive second-level tests, as recently demonstrated in the case of anti-LC1 antibodies that can be negative by standard indirect immunofluorescence but easily detected by immunoblotting [22].

The most consistent histological findings in AAIH are lymphoplasmacytic infiltrate, centrilobular necrosis, and interface hepatitis. Sometimes, only centrilobular necrosis is observed to support the diagnosis [9, 23–25]. In our study, 82% had interface hepatitis, and 81% had chronic hepatitis. Other classical findings of AIH were infrequent; specifically, rosettes were present only a in few patients (36%). Interestingly, cholestasis was present in only 41% of patients but in all of the patients who died or received LT (see below). Most of the patients (63%) had mild fibrosis (*F*0/*F*1), which may reflect a more recent disease onset; in contrast, advanced fibrosis (*F*3/*F*4) was present in 27% of patients and was probably related to the reactivation of an undiagnosed chronic disease [6].

Taken together, clinical characteristics and laboratory and histological findings help to establish the diagnosis of AIH so that appropriate therapy can then be indicated. In fact, the standard treatment for AIH (steroids alone or in combination with azathioprine) induces remission of the disease in more than 80% of patients [26, 27]. However, achieving these results in patients with an acute presentation of AIH is more complex because early diagnosis is difficult and treatment initiation is challenging. Although validated AIH scores are not suitable for diagnostic purposes in an acute setting [7], they are used as a diagnostic method in clinical practice [12]. In our cohort of patients with confirmed AAIH, the original and simplified scores showed poor performances for a definitive diagnosis of AIH and identified 53 and 66% of the patients, respectively. This finding is similar to the result described in a recent Italian study, in which the simplified score for definite AIH only identified 46% of the patients with AAIH [28]. This phenomenon is possibly related to features unique to the acute presentation, such as serological autoantibody negativity, normal IgG levels in a high percentage of patients, and atypical histological findings [6, 10, 11]. For the aforementioned reasons, these scores must be used cautiously in AAIH, and new scoring systems should be developed.

In our study, the occurrence of in-hospital mortality or LT was low (7.9%). Even though cases with more severe presentations were higher than in previous studies (11% vs. 3–6% of fulminant hepatitis) [4–6], mortality and the need for LT were lower [8–11]. A decade of advancement since previous research was conducted as well as racial differences are some potential explanations. Additionally, we included a high percentage of patients with nonsevere AAIH, a group expected to have a lower mortality rate. Interestingly, all patients who died or underwent transplantation had fulminant hepatitis and histological cholestasis. Because of the latter, neither fulminant presentation nor cholestasis was included in the regression analysis, as both were associated with the outcome, which could also explain the differences between this study and other studies. Additionally, if AAIH is suspected, (i) liver biopsy is mandatory and (ii) fulminant hepatitis or histological cholestasis should be considered an indication for recommending the patient to an LT center.

Similar to patients with other forms of acute hepatitis, in our series of patients with AIH, bilirubin, and PT are regarded as risk factors for adverse outcomes. In previous reports, histological cholestasis was described as a feature of more severe forms of AIH [11], consistent with its association with a poor outcome in our study. Similarly, histological and biochemical cholestasis are related to worse outcomes for patients with many liver diseases, such as alcoholic hepatitis [29] or drug-induced liver injury [30], as observed in our series. To the best of our knowledge, this is one of the first cohorts with more than 100 patients reporting risk factors for death/LT in AAIH, but due to the limited number of events, we cannot know if all these risk factors are independent of each other.

Corticosteroids are usually indicated for patients with AAIH. Although observational studies have associated corticosteroid use with a lower disease relapse rate [31], no randomized trials have proven corticoid efficacy against AAIH [32]. According to our results, this treatment was indicated for a high number of patients (94%). Furthermore, corticosteroid use was associated with lower odds of in-hospital death or LT. However, the latter result should be interpreted with caution since sicker patients who are candidates for transplantation usually do not receive steroids because of their negligible effects [26]. Patients with severe or fulminant acute hepatitis were treated less often with corticosteroids (OR = 0.088, *p* value = 0.026). Therefore, confounding by indication is likely to present in the reported association and should not be viewed as a causal effect [33].

The main limitations of our study derive from its retrospective and observational design. Longer follow-up could have provided more information on long-term survival; however, many patients were lost to follow-up since many patients returned to their homeland after the initial diagnosis and treatment of AIH at one of our referral centers. The number of events prevented us from including more independent variables in multivariate regression analyses, limiting the interpretation of the reported associations as causal effects. Finally, this study was conducted only with Chilean patients, which confers a limitation, as regional differences in the AIH patient profiles exist due to different genetic backgrounds; therefore, the results might not be the same for patients from different regions/countries [34]. Multicenter multiregional studies should be conducted to assess this source of bias. However, this is one of the most extensive multicenter reports of patients with AAIH, providing a complete description of the clinical, laboratory, and histological characteristics and the risk factors for robust outcomes.

In this large cohort of patients with AAIH, most were middle-aged and female, with specific laboratory and histological characteristics that differed from those reported in patients with chronic AIH. Notably, AIH scoring systems were negative in 30% of patients, highlighting the need for specific scores for acute presentation. Fulminant hepatitis, histological cholestasis, alkaline phosphatase, bilirubin, and prothrombin were associated with death/LT. Further studies are necessary to replicate our results while controlling for potential confounding factors.

## Figures and Tables

**Table 1:** Basal characteristics of the patients according to disease severity.

Characteristics	Nonsevere (*N* = 79)	Severe (*N* = 33)	Fulminant (*N* = 14)	*p* value
Age, years	50.6 ± 14.5	45.0 ± 16.5	49.1 ± 15.8	0.213
Female sex	60 (76.0%)	24 (73.0%)	13 (93.0%)	0.337
Autoimmune comorbidities	19 (24.0%)	9 (27.0%)	1 (7.0%)	0.337
Hypothyroidism	12 (15.0%)	6 (18.0%)	1 (7.0%)	0.756
Sjögren disease	4 (5.0%)	4 (12.0%)	0 (0.0%)	0.271
TP (idiopathic)	2 (3.0%)	0 (0.0%)	0 (0.0%)	—
Ulcerative colitis	1 (1.0%)	1 (3.0%)	0 (0.0%)	—
Others	3 (4.0%)	0 (0.0%)	0 (0.0%)	—
Total bilirubin—(mg/dL)	6.0 (1.7–11.9)	15.3 (9.0–22.3)	20.6 (15.5–28.8)	<0.001
AST—(U/L)	1016 (635–1381)	1240 (948–1864)	1094 (664–2000)	0.050
ALT—(U/L)	1227 (818–1818)	1178 (775–1478)	902 (602–2102)	0.649
AP—(U/L)	209 (156–283)	231 (191–313)	273 (186–498)	0.075
GGT—(U/L)	295 (189–429)	216 (155–347)	174 (112–478)	0.154
Prothrombin %	74.3 ± 12.9	42.3 ± 6.4	30.9 ± 13.1	<0.001
ANA > 1 : 40	60 (76.0%)	23 (70.0%)	10 (71.0%)	0.753
SMA > 1 : 40	38 (48.0%)	15 (45.0%)	7 (50.0%)	0.965
AMA (above normal)	4 (5.0%)	2 (6.0%)	0 (0.0%)	1.000
Immunoglobulin G				
Above normal	57 (72.2%)	24 (72.7%)	7 (50.0%)	0.435
No data	3 (3.8%)	1 (3.0%)	1 (7.1%)	
Anti-LKM1				
Above normal	4 (5.0%)	0 (0.0%)	0 (0.0%)	0.170
No data	2 (1.6%)	2 (1.7%)	0 (0.0%)	
Corticosteroids use	78 (99.0%)	33 (100.0%)	8 (57.0%)	<0.001
Combined outcome	0 (0.0%)	0 (0.0%)	10 (71.4%)	<0.001
Death	0 (0.0%)	0 (0.0%)	4 (28.6%)	<0.001
Liver transplant	0 (0.0%)	0 (0.0%)	9 (64.3%)	<0.001

Numerical data are presented as the mean ± DS or median (IQR) depending on their distribution. LT: liver transplant; AST: aspartate aminotransferase; ALT: alanine aminotransferase; AP: alkaline phosphatase; GGT: gamma-glutamyl transferase; ANA: antinuclear antibody; SMA: smooth muscle antibody; AMA: antimitochondrial antibody; anti-LKM1: anti-liver-kidney microsome antibodies.

**Table 2:** Basal characteristics of the patients with acute autoimmune hepatitis.

Characteristics	Total (*N* = 126)	No Death/LT (*N* = 116)	In-hospital Death/LT (*N* = 10)	*p* value
Age, years	49.0 ± 15.3	48.4 ± 15.4	55.7 ± 12.0	0.150
Female sex (%)	97 (77.0%)	88 (75.9%)	9 (90.0%)	0.452
Autoimmune comorbidities (%)	29 (23.0%)	28 (24.1%)	1 (10.0%)	0.452
Hypothyroidism	19 (15.1%)	18 (15.5%)	1 (10.0%)	—
Sjögren disease	8 (6.4%)	8 (6.9%)	0 (0.0%)	—
TP (idiopathic)	2 (1.6%)	2 (1.7%)	0 (0.0%)	—
Ulcerative colitis	2 (1.6%)	2 (1.7%)	0 (0.0%)	—
Others	3 (2.4%)	3 (2.6%)	0 (0.0%)	—
Total bilirubin—(mg/dL)	10.0 (3.4–17.9)	8.6 (3.0–15.8)	20.0 (15.5–28.8)	<0.001
AST—(U/L)	1042 (678–1469)	1042 (685–1461)	958 (664–2000)	0.935
ALT—(U/L)	1171 (768–1740)	1201 (797–1738)	815 (602–3000)	0.542
AP—(U/L)	216 (169–310)	215 (168–298)	273 (212–594)	0.127
GGT—(U/L)	270.0 (159–406)	269 (160–389)	317 (119–510)	0.815
Prothrombin %	61.1 ± 20.9	63.4 ± 19.7	33.7 ± 14.3	<0.001
ANA > 1 : 40 (%)	93 (73.8%)	86 (74.1%)	7 (70.0%)	0.721
SMA > 1 : 40 (%)	60 (47.6%)	55 (47.4%)	5 (50.0%)	1.000
AMA (above normal) (%)	6 (4.8%)	6 (5.2%)	0 (0.0%)	—
Immunoglobulin G (%)				
Above normal	88 (69.8%)	84 (72.4%)	4 (40.0%)	0.069
No data	5 (4.0%)	4 (3.5%)	1 (10.0%)	
Anti-LKM1 (%)				
Above normal	4 (3.2%)	4 (3.5%)	0 (0.0%)	—
No data	2 (1.6%)	2 (1.7%)	0 (0.0%)	
Disease severity (%)				
Nonsevere	79 (62.7%)	79 (68.1%)	0 (0.0%)	<0.001
Severe	33 (26.2%)	33 (28.4%)	0 (0.0%)	
Fulminant	14 (11.1%)	4 (3.4%)	10 (100.0%)	
Corticosteroids use (%)	119 (94.4%)	113 (97.4%)	6 (60.0%)	<0.001

Numerical data are presented as the mean ± DS or median (IQR) depending on their distribution. LT: liver transplant; AST: aspartate aminotransferase; ALT: alanine aminotransferase; AP: alkaline phosphatase; GGT: gamma-glutamyl transferase; ANA: antinuclear antibody; SMA: smooth muscle antibody, AMA: antimitochondrial antibody; anti-LKM1: anti-liver-kidney microsome antibodies.

**Table 3:** Histological findings from liver biopsies of acute autoimmune hepatitis patients.

Histological finding	Total (*N* = 126)	No Death/LT (*N* = 116)	In-hospital Death/LT (*N* = 10)	*p* value
Interface hepatitis	103 (81.7%)	96 (82.8%)	7 (70%)	0.388
Plasma cells	109 (86.5%)	100 (86.2%)	9 (90.0%)	1.000
Chronic hepatitis	102 (81.0%)	95 (81.9%)	7 (70.0%)	0.401
Rosettes	45 (35.7%)	42 (36.2%)	3 (30.0%)	1.000
Cholestasis	52 (41.3%)	42 (36.2%)	10 (100.0%)	<0.001
Centrilobular necrosis				
Mild	51 (40.5%)	48 (41.4%)	3 (30.0%)	0.119
Moderate	23 (18.3%)	23 (19.8%)	0 (0.0%)	
Severe	52 (41.3%)	45 (38.8%)	7 (70.0%)	
Fibrosis (METAVIR)	1 (0–3)	1 (0–3)	1 (0–3)	0.831
Mod/Severe†	34 (27.0%)	31 (26.7%)	3 (30.0%)	1.000

†: Moderate/severe fibrosis is defined as a METAVIR score of *F*3 or *F*4.

**Table 4:** Characteristics of the deceased patients.

	Patient 1	Patient 2	Patient 3	Patient 4
Age	59	49	68	72
Sex	Male	Female	Female	Female
AIH presentation	Fulminant	Fulminant	Fulminant	Fulminant
Chronic hepatitis	Yes	No	No	Yes
Necrosis	No	No	Severe	No
Fibrosis	*F*4	*F*4	*F*1	*F*1
Treatment with steroids	Yes	No	No	Yes
Liver transplantation	Yes	Yes	Yes	No
Total bilirubin—(mg/dL)	30.3	28.8	7.2	17.5
AST/ALT—(U/L)	378/433	863/602	9547/4276	1847/1012
Immunoglobulin G—(mg/dL)	1900	1070	1530	2450
Prothrombin—(%)	26	40	48	44
MELD	30	25	19	23
ANA	1/640	1/80	1/80	1/640
SMA	1/640	1/320	(—)	1/320
Cause of death	Septic shock	Septic shock	Septic shock	Septic shock

AST: aspartate aminotransferase; ALT: alanine aminotransferase; ANA: antinuclear antibody; SMA: smooth muscle antibody; MELD: model of end-stage liver disease.

**Table 5:** Risk factors for adverse outcomes in acute autoimmune hepatitis patients.

Risk factor	Unadjusted OR (95% CI)	*p* value	Adjusted OR^*∗*^ (95% CI)	*p* value
Alkaline phosphatase	1.002 (1.000–1.004)	0.046	1.003 (1.001–1.006)	0.019
Total bilirubin	1.144 (1.055–1.240)	0.001	1.108 (1.012–1.213)	0.027
Prothrombin time ^†^	26.677 (2.181–53.862)	0.003	13.164 (1.515–114.421)	0.019
Corticosteroid use	0.040 (0.007–0.220)	< 0.001	0.095 (0.015–0.606)	0.013

Only variables with a *p* value < 0.05 for crude odds ratio (OR) are shown. a: less than 50%. ^†^: adjusted for the prothrombin time or bilirubin level (for the case of prothrombin).

**Table 6:** Histological findings from liver biopsies according to disease severity.

Histological finding	Nonsevere (*N* = 79)	Severe (*N* = 33)	Fulminant (*N* = 14)	*p* value
Interface hepatitis	67 (85.0%)	25 (76.0%)	11 (79.0%)	0.424
Plasma cells	70 (89.0%)	26 (79.0%)	13 (93.0%)	0.388
Chronic hepatitis	66 (84.0%)	25 (76.0%)	11 (79.0%)	0.625
Rosettes	27 (34.0%)	15 (45.0%)	3 (21.0%)	0.297
Cholestasis	23 (29.0%)	16 (48.0%)	13 (93.0%)	<0.001
Centrilobular necrosis				
Mild	39 (49.0%)	7 (21.0%)	5 (36.0%)	<0.001
Moderate	19 (24.0%)	4 (12.0%)	0 (0.0%)	
Severe	21 (27.0%)	22 (67.0%)	9 (64.0%)	
Fibrosis (METAVIR)	1 (0–3)	1 (0–3)	1 (0–3)	1.000
Mod/Severe^†^	21 (27.0%)	9 (27.0%)	4 (29.0%)	1.000

^†^: Moderate/severe fibrosis is defined as a METAVIR score of *F*3 or *F*4.

## Data Availability

The data used to support the findings of this study are available from the corresponding author upon request.

## Abbreviations

AIH: Autoimmune hepatitis
AAIH: Acute AIH
I_g_G: Immunoglobulin G
LT: Liver transplantation
ALT: Alanine aminotransferase
AST: Aspartate aminotransferase
GGT: Gamma-glutamyl transferase
AP: Alkaline phosphatase
PT: Prothrombin time
INR: International normalized ratio
ANA: Antinuclear antibody
SMA: Smooth muscle antibody
OR: Odds ratio.

## Additional Points

(i) Typical serological and histological features of chronic AIH may be absent in AAIH. (ii) The most severe form of presentation (fulminant hepatitis) and the presence of cholestasis in the liver biopsy are associated with adverse outcomes (liver transplantation and in-hospital death). (iii) If AAIH is suspected, whenever possible, a liver biopsy should be performed. Fulminant hepatitis or histological cholestasis must alert the patient to an LT center.
